# Longitudinal Metabolic Biomarker Profile of Hyperketonemic Cows from Dry-Off to Peak Lactation and Identification of Prognostic Classifiers

**DOI:** 10.3390/ani11051353

**Published:** 2021-05-10

**Authors:** Anna Mareike Couperus, Fabian Schroeder, Peter Hettegger, Johann Huber, Thomas Wittek, Johannes R. Peham

**Affiliations:** 1 Molecular Diagnostics, Center for Health and Bioresources, AIT Austrian Institute of Technology, 1210 Vienna, Austria; p.hettegger@gmail.com (P.H.); johannes.peham@ait.ac.at (J.R.P.); 2 Institute of Statistics and Mathematical Methods in Economics, Vienna University of Technology, 1040 Vienna, Austria; Fabian.Schroeder@tuwien.ac.at; 3 University Clinic for Ruminants, University of Veterinary Medicine Vienna, 1210 Vienna, Austria; Johann.Huber@vetmeduni.ac.at (J.H.); Thomas.Wittek@vetmeduni.ac.at (T.W.)

**Keywords:** hyperketonemia, ketosis, subclinical ketosis, progression, longitudinal, biomarker monitoring, metabolic profile, beta-hydroxybutyrate, prognostic biomarker

## Abstract

**Simple Summary:**

Over the last decades, the dairy industry has primarily been focused on constantly increasing milk yields per cow. Consequently, the incidence of metabolic disorders is rising. In this study, we investigate the effect of hyperketonemia on the longitudinal progression of four metabolic biomarkers in dairy cows and possible predictive biomarkers for hyperketonemia. Our findings provide new insights into the metabolic challenges of dairy cows, and we propose novel approaches enabling an early onset diagnosis of hyperketonemia.

**Abstract:**

Currently about 30% to 50% of all dairy cows are affected by a metabolic or infectious disease during the transition period. A key factor for preventive actions is the ability to precisely predict metabolic diseases at an early stage. We report the longitudinal metabolic profile of non-esterified fatty acids, beta-hydroxybutyrate (BHB), total bilirubin, and aspartate aminotransferase in hyperketonemic dairy cows. Aiming for a novel measurement regime to improve metabolic health in dairy cows, we evaluated prognostic classifiers for hyperketonemia. In the observational longitudinal study, 99 healthy adult primiparous and multiparous Simmental dairy cows were included. Every cow was monitored weekly for 14 consecutive weeks, beginning two weeks prior to the expected day of parturition until peak lactation. Cows with serum concentrations of BHB > 0.8 mmol/L were considered hyperketonemic. Biomarker profiles were fitted by the maximum likelihood method using a mixed effects natural cubic spline model. In the hyperketonemic group, the BHB profile remained significantly higher than that of the control group until the end of the study period. As a prognostic classifier, the cut-off level of 0.54 mmol/L BHB measured on the 10th day post partum had the highest area under the curve. These results provide new longitudinal insights into the metabolic biomarker progression of dairy cows and enable an early onset diagnosis of hyperketonemia.

## 1. Introduction

In recent decades, the dairy industry has been focused on constantly increasing milk yields. The high average milk yield per cow was mostly achieved by genetic selection in combination with optimized farm management and feeding strategies [1]. Moreover, Rauw et al. [2] claim that production traits such as milk yields are twice as heritable as metabolic traits (e.g., feed intake). High average milk yields and high metabolic rates contribute to an extended and more intense negative energy balance (NEB) around calving. NEB has been known to increase the risk for several metabolic diseases and infections [3,4,5,6]. LeBlanc [7] claims that 30% to 50% of dairy cows are affected by some form of metabolic or infectious disease during the transition period from dry-off to early lactation. One of the most important metabolic disorders in dairy cows is ketosis. Ketosis is defined by an elevated concentration of ketone bodies in blood. Ketone bodies are produced in the mitochondria of hepatocytes and are a part of the normal adaptive metabolic response. The three ketone bodies are beta-hydroxybutyrate (BHB), acetoacetate, and acetone. BHB is the most stable form in blood and is commonly used for diagnostics. Different cut-off levels for BHB have been published. Fürll [8] recommended a cut-off level for BHB of 0.62 mmol/L indicating hyperketonemia. BHB concentrations above 1.2 mmol/L have been defined as subclinical ketosis (SCK) [9]. SCK is associated with an increased risk of numerous diseases and infections such as displaced abomasum (DA), uterine infection, and mastitis [10,11,12]. Further, SCK is suspected to impair fertility and reduce milk yield [13,14]. Van Saun and Sniffen [15] estimate the median incidence risk of SCK at 53%, compiled over several studies. McArt, Nydam, and Overton [16] estimate the average total cost of one SCK case at USD 375 and USD 256 for primiparous and multiparous cows, respectively. BHB concentrations above 3 mmol/L are classified as clinical ketosis [17]. For clinical ketosis Kelton et al. [18] reported a 4.8% median incidence risk compiled over several studies. To reflect the effect of ketosis on energy metabolism, additional biomarkers need to be considered. The recommended biomarker panel for fat cow syndrome (FCS) or hepatic lipolysis consists of non-esterified fatty acids (NEFA), BHB, total bilirubin (tBIL), and aspartate aminotransferase (AST) [19]. FCS similar to ketosis develops due to an imbalance in the energy metabolism [13]. During FCS, the hepatic uptake of metabolized lipids exceeds their oxidation and lipid exportation from the liver. Consequently, excess lipids are accumulated as triacylglycerol in the liver, compromising its metabolic performance. NEFA is a metabolite during lipolysis and thereby an indicator of fat mobilization and NEB [20]. BHB as discussed above is a ketone body and a marker for energy metabolism. tBIL is a product of hemoglobin catabolism and biliary metabolized. An elevated concentration is related to a decreased bile flow and impaired liver function and is a marker for hepatocyte dysfunction and damage [21]. Furthermore, tBIL is positively correlated to NEFA due to their transport concurrence. AST activity serves as a marker for cell integrity. Increased serum activity is caused by damaged tissue and hepatic lesions [8,19,22].

The aim of our study was to investigate and compare the long-term metabolic biomarker profile of cows with hyperketonemia and non-ketotic cows. Further, we wanted to identify novel prognostic classifiers for hyperketonemia and derive a cut-off level to avoid hyperketonemia. Our main hypothesis was that a persisting change in the biomarker course already occurs in mild forms of hyperketonemia. Further, we emphasize that the definition of cut-off points should rather be based on maintaining and nurturing health than on production yield and impairment of health. The ability to predict the risk and classify metabolic diseases in dairy cows early on is a key factor in taking effective preventive actions.

## 2. Materials and Methods

### 2.1. Animals, Study Design, Housing and Diets

A longitudinal observational study design was chosen. The study was performed at the teaching and research dairy farm of the Vetmeduni Vienna (VetFarm Kremesberg, Pottenstein, Austria) between April 2016 and December 2017. In total, 104 healthy adult primiparous (number of samples (*n*) = 27) and multiparous (*n* = 74) Simmental dairy cows were enrolled in this study. During the study period, 2 cows died due to birth complications, 1 cow was euthanized due to toxic mastitis, 7 cows were sold during the study trial, and 1 cow was excluded due to repeated dangerous behavior during blood sampling. Cows with more than 10 observations were considered for the final data set (1256 observations from 99 cows). Lactating cows were fed a total mixed ration consisting of 19.23% hay, 16.67% grass-silage, 44.23% corn silage, 11.41% grain mix, and 8.46% protein supplement (RINDASTAR 39 XP; H. Wilhelm Schaumann GmbH & Co KG, Brunn am Gebirge, Austria) based on dry matter basis. Dry cows were fed with a dry-cow total mixed ration consisting of 24.25% hay, 9.33% grass silage, 46.08% corn silage, 12.13% barley straw, and 8.21% protein supplement (RINDASTAR 39 XP; H. Wilhelm Schaumann GmbH & Co KG, Brunn am Gebirge, Austria) based on dry matter basis. The mixed ration had a net energy lactation of 6.7 MJ/kg and a metabolizable energy of 10.4 MJ/kg. The composition of the grain mixture varied to a minor degree throughout the seasons. Feed was mixed and delivered by an automatic mixing and feeding system (Triomatic T15, Trioliet Feeding Technology, Oldenzaal, The Netherlands). Fresh feed was offered to lactating cows ad libitum eleven times per day (between 4:50 a.m. and 9:30 p.m.). Dry cows were offered fresh feed ad libitum twice per day (7:15 a.m. and 4.00 p.m.). All cows were housed in a free stall barn with straw bedding and had ad libitum access to water and mineral stones (Raiffeisenverband Salzburg reg. Gen.m.b.H, Salzburg, Austria) throughout the study. Twice a day (6:30 a.m. and 4:30 p.m.), the lactating cows were milked in a 4 × 4 tandem milking parlor (DeLaval GmbH Eugendorf, Austria). 

### 2.2. Data Collection

Every cow was monitored for 14 consecutive weeks. Sampling was performed once per week after morning milking approximately 2 to 3 h after first feeding. The first sample was taken 2 weeks prior to the expected day of birth.

#### 2.2.1. Blood Sampling

The blood was collected from the jugular vein by vacuum tube system (Vacuette^®^, Greiner Bio-one International, Kremsmünster, Austria) using 10 mL serum vacutainer tubes with coagulant (Vacuette^®^ Z Serum Clot Activator, Greiner Bio-one International, Kremsmünster, Austria). The blood samples were kept at room temperature for 2 h to allow for clotting. The serum was separated by centrifugation at 3000× *g* for 15 min. Samples were stored at −80 °C until analysis, which was performed within a maximum of 8 weeks.

#### 2.2.2. Herd Monitoring

Herd monitoring data was provided by VetFarm Kremesberg. Routine point of care BHB measurements were performed 7 days p.p., wherein coccygeal venous blood samples were analyzed by an electronic hand-held device (Freestyle Precision, Abbott Ges.m.b.H., Vienna, Austria). BHB concentrations above 1 mmol/L were considered as positive ketosis samples, and affected cows were treated with monopropylene glycol (PG) and glycerin (PropyLac^®^, Garant Tiernahrung Ges.m.b.H, Pöchlarn, Austria) as food supplement for a week. Treatment was started after the sample for the study was withdrawn. In total, 16 out of 1256 samples were drawn after a weekly treatment.

### 2.3. Serum Analysis

In all serum samples, tBIL, BHB, NEFA, and AST activity were analyzed with an autoanalyzer for clinical chemistry (Cobas 6000/c501; Roche Diagnostics GmbH, Vienna, Austria) using standardized colorimetric enzymatic assays. NEFA was analyzed with the ASC-ACOD method (Wako Chemicals, Richmond, VA, USA, inter-day coefficient of variability (CV) < 0.75%, inter-day CV at 0.55 mmol/L = 0.75%, inter-day CV at 1.08 mmol/L = 4.91%). BHB was determined using the Ranbut method (Randox Laboratories Ltd., London, UK, inter-day CV = 0.57%, intra-day CV = 0.99%). tBIL was measured with the Bilirubin Total DPD Gen.2 Kit (Roche Diagnostics GmbH, Vienna, Austria, inter-day CV = 1.6%, intra-day CV = 2.6%). AST activity was analyzed by kinetic measurement of the enzyme activity with pyridoxal phosphate activation recommended by the International Federation of Clinical Chemistry (Roche Diagnostics GmbH, Vienna, Austria, inter-day CV = 0.6%, intra-day CV = 0.8%). All measurements were performed at the Clinical Pathology Platform of the Vetmeduni Vienna.

Samples with BHB concentrations > 0.8 mmol/l were classified as hyperketonemic. The cut-off was set in between the tolerance cut-off for individuals (0.62 mmol/L) recommended by Fürll et al. [23] and the cut-off used by the herd monitoring team (1.00 mmol/L). Further, Fürll [8] associates a BHB level > 0.85 mmol/L on the 3rd day p.p. in combination with other parameters as an indicator for increased fat mobilization and FCS. Cows with samples of BHB concentrations ≤ 0.8 mmol/L were selected as control group. SCK was defined as a BHB concentration > 1.2 mmol/L [24,25] and cows with samples of BHB concentrations ≤ 1.2 mmol/L were considered as control group. 

### 2.4. Statistical Analysis

All data were analyzed with R (Version 3.0.4). A first distribution of hyperketonemia and SCK over the study period was determined by an empirical cumulative distribution function. Overall hyperketonemia and SCK incidence were calculated by dividing the number of cows with hyperketonemia or SCK by the total number of cows tested. Further, the time span in days between last sampling ante partum (a.p.) to partum was calculated for each individual. The maximum calculated time span marks the smallest possible time interval for further classification analysis (e.g., every other day or weekly measurements). Using the maximum time span in classification models assures that at least one measurement point of each tested cow lies within the chosen time interval. The normality of all biomarkers (response variables) was tested with the Shapiro–Wilk test, with all variables requiring Log-transformation. A mixed effects natural cubic spline model was chosen to fit the course of hyperketonemia, SCK, and control cases over lactation stages. The model was fit to the data between 27 days a.p. to 80 days p.p. A family of splines with a degree of 8 (n = 8) was defined, and the optimal number of splines for the final fit was chosen based on the Akaike information criterion (AIC). The resulting spline function was smoothly joined at a fixed number of knots. The number of knots was defined by K = *n* − 1 (K = 7). The positions of the knots (20 days a.p., 7 days a.p., day of parturition, 7 days p.p., 14 days p.p., 25 days p.p. and 50 days p.p.) were chosen at time points, where structural changes were expected, while maintaining a uniform distribution of all knots throughout the fitted time span. Data obtained from the same cow were considered as repeated measurements. The final model was built in 3 stages. In the first stage a mixed effects model with solely a random intercept was fitted by maximum likelihood. In the second stage, hyperketonemia was added as a fixed effect to the spline coefficient to look for an overall effect of hyperketonemia. Finally, in the third stage hyperketonemia was additionally included as a random effect to the spline coefficient to evaluate a possible interaction of hyperketonemia and individuals. As described by Durrleman and Simon [26], spline functions are linear in the regression coefficients. Hence, the significance of the introduced covariates was evaluated by comparing all 3 models using a one-way ANOVA. To test if the biomarker concentration between hyperketonemic, SCK, and control cows differed significantly during the study period, one-sample *t*-tests were performed. The study period was subdivided into intervals of 6 days, starting at 15 days a.p. until 62 days p.p. A *t*-test was performed for all time intervals, t0, and t1 for each biomarker. The *t*-test was performed as a descriptive analysis; therefore, we did not correct for multiple measurements. As potential classifiers for hyperketonemia we considered the following features: (I) the median biomarker concentration a.p. (baseline), (II) the maximum biomarker value a.p., (III) biomarker concentration at partum (t0), (IV) daily biomarker concentration on the first day until the 10th day p.p. (t1), (V) biomarker increase from baseline to t1, (VI) biomarker increase from partum to t1, and (VII) biomarker concentration within different lactation. If measurements were not conducted on the exact time points, corresponding biomarker concentrations were linear interpolated using their nearest neighbor. All classifiers were tested by row-wise calculation of receiver operating characteristic (ROC) curves including their corresponding area under the curve (AUC). Single classifiers and random decision trees were evaluated. As criteria for the random decision trees, a minimum of 10 observations at each tree node and 5 cross validations were selected and analyzed using the R package “rpart”. Cut-off values were calculated by the criterion based on the Youden’s index using the R package “OptimalCutpoints”. The cost value of false negative (CFN) cases was set to 4 to reflect the impact of a false negative versus a false positive case. A false negative and thereby potentially missed ketosis case would cost more and has a higher impact than supplementing a false positive cow with PG and glycerin. The classifier achieving the highest AUC was presented. Additional data are shown in Appendix A.

## 3. Results

**Figure 1:**
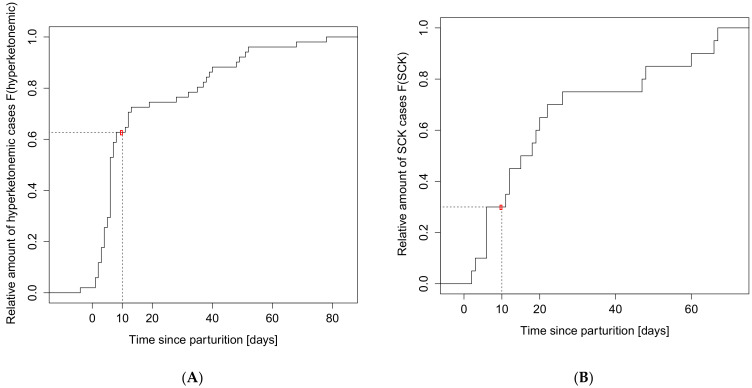
The relative distribution function of cases during the study in relation to the day of parturition: (**A**) Relative distribution function of hyperketonemic cases during the study (BHB > 0.8 mmol/L) in relation to the day of parturition. On the 6th day post partum (p.p.), 37% of cases progressing to hyperketonemia were undiagnosed. On the 10th day p.p., 37% of future hyperketonemia cases were undiagnosed; (**B**) Relative distribution function of subclinical ketosis (SCK) cases during the study (BHB > 1.2 mmol/L) in relation to the day of parturition. The highest leap of cases occurred on the 6th day post partum (p.p.). Until the 10th day p.p., 30% of the SCK cases were diagnosed.

In this study, 52% of the cows had at least one BHB test result higher than 0.8 mmol/L during the study and were classified as hyperketonemic. The cumulative distribution function of hyperketonemia cases in relation to the day of parturition is presented in Figure 1A. On the day of parturition, 2% of cows were hyperketonemic. On the 6th day p.p., the highest leap of cases (from 29% to 53%) was observed. Before the 10th day p.p. the case number elevated to 63%. A second leap, from 77% to 92% of cases, was observed between 30 to 50 days p.p. Furthermore, this means that 47% (measurement at 6th day) and 37% (measurement at 10th day) of cases progressing to hyperketonemia were not detected with a single-day measuring scheme. In the present study the overall SCK incidence was 20%. The cumulative distribution function of SCK cases in relation to the day of parturition is shown in Figure 1B. 

**Figure 2:**
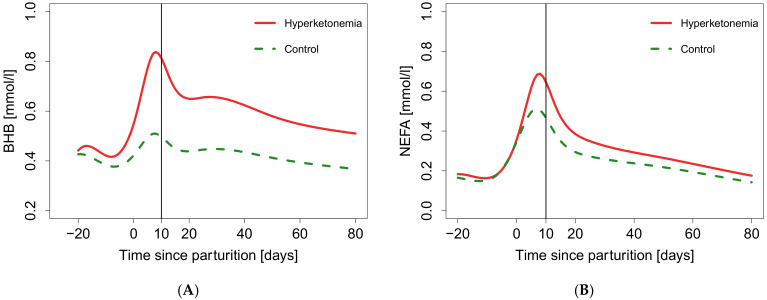

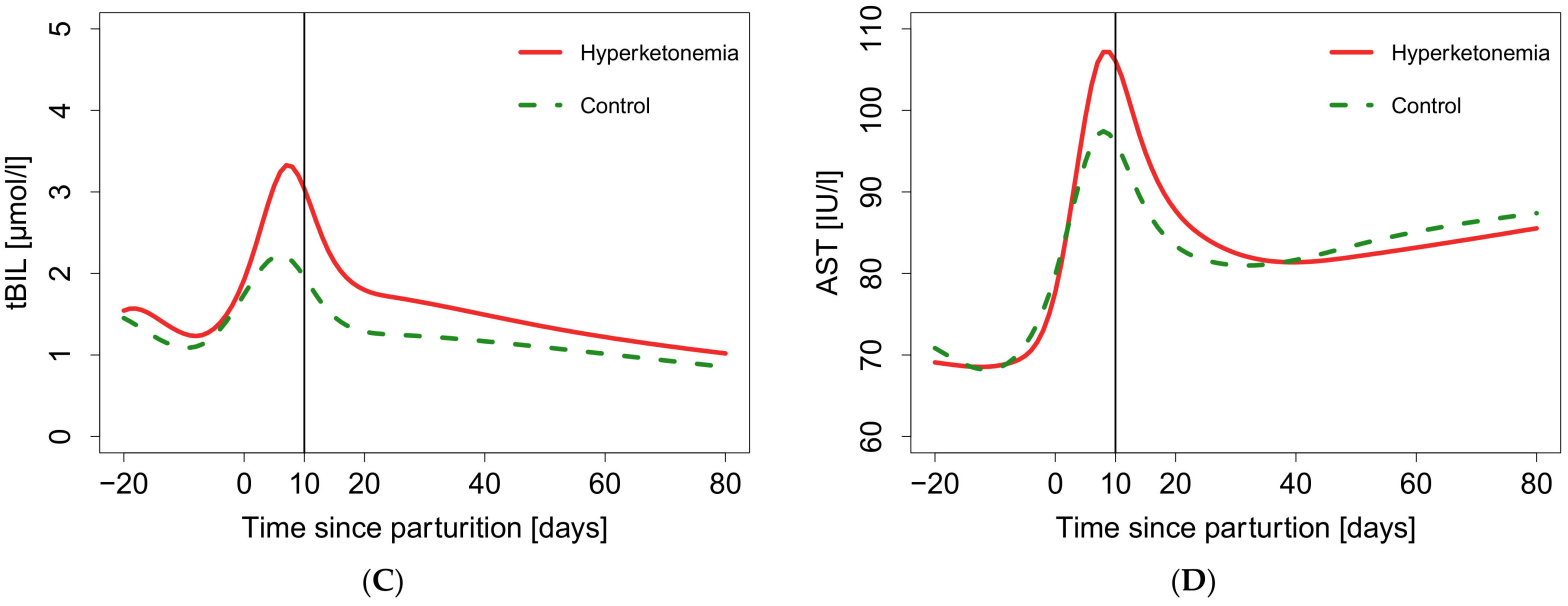
Natural cubic spline fit of biomarkers for hyperketonemia (BHB > 0.8 mmol/L) and control (BHB ≤ 0.8 mmol/L) group between 20 days ante partum and 80 days post partum. The 10th day p.p. is indicated by a vertical line: (**A**) Natural cubic spline fit of beta-hydroxybutyrate (BHB); (**B**) Natural cubic spline fit of non-esterified fatty acids (NEFA); (**C**) Natural cubic spline fit of total bilirubin (tBIL); (**D**) Natural cubic spline fit of aspartate aminotransferase (AST).

The final natural cubic spline fits of BHB (Figure 2A), NEFA (Figure 2B), tBIL (Figure 2C), and AST (Figure 2D) of hyperketonemia are shown. The spline fits represent a BHB course for an individual over time. Hyperketonemia had a significant effect on the time course of all tested biomarkers. For BHB the log likelihood ratio of the final model was χ^2^(17) = 48.06, *p* < 0.001. Two peaks of BHB concentration were observed throughout the fitted period. The first peak occurred directly after parturition, whereas the second occurred between 20 to 40 days p.p. Furthermore, the BHB concentration in the hyperketonemic group remained higher compared to the control group until 80 days p.p. For NEFA the log likelihood ratio of the final model was χ^2^(17) = 115.63, *p* < 0.001. In the hyperketonemic group, one peak of NEFA concentration was observed directly after parturition followed by a continuous decline until the end of the fitted period. In the control group, two peaks occurred, wherein the first was seen after parturition and the second between 20 to 40 days p.p. For tBIL the log likelihood ratio of the final model was χ^2^(17) = 86.58, *p* < 0.001. In both groups one peak of tBIL concentration was observed directly after parturition. For AST the log likelihood ratio of the final model was χ^2^(17) = 166.62, *p* < 0.001. In both groups one peak of AST concentration was observed directly after parturition. Subsequently, AST levels declined in both groups. After 30 days p.p., AST concentration steadily increased in the remaining study period. The BHB concentration was significantly higher in hyperketonemic cows than in the control group (*p* < 0.5) from 9 days a.p. to 62 days p.p. Detailed results of time intervals showing significantly higher biomarker concentrations are presented in Appendix A.

**Figure 3:**
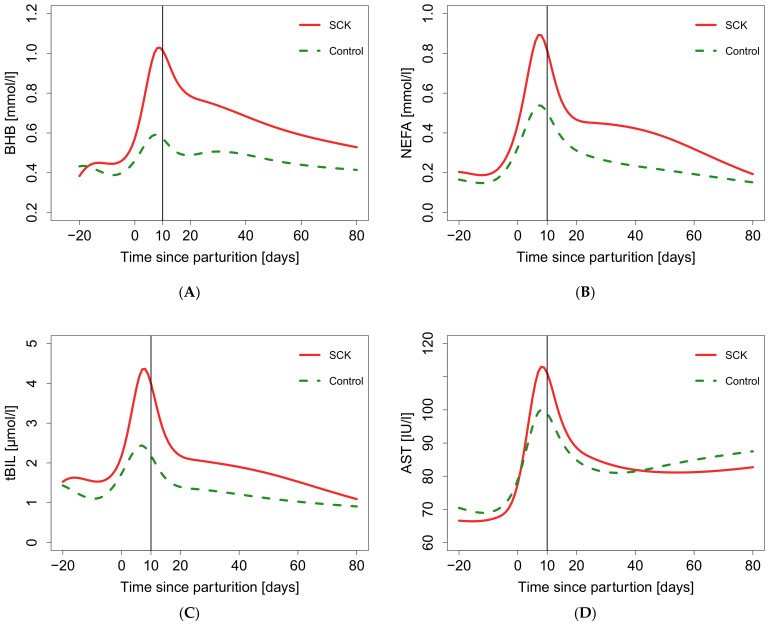
Natural cubic spline fit of biomarkers for subclinical ketosis (SCK) (BHB > 1.2 mmol/L) and control (BHB ≤ 1.2 mmol/L) group between 20 days ante partum and 80 days post partum. The 10th day p.p. is indicated by a vertical line: (**A**) Natural cubic spline fit of beta-hydroxybutyrate (BHB); (**B**) Natural cubic spline fit of non-esterified fatty acids (NEFA); (**C**) Natural cubic spline fit of total bilirubin (tBIL); (**D**) Natural cubic spline fit of aspartate aminotransferase (AST).

The final natural cubic spline fits of BHB (Figure 3A), NEFA (Figure 3B), tBIL (Figure 3C), and AST (Figure 3D) of SCK are shown. The spline fits represent a BHB course for an individual over time. SCK had a significant effect on the time course of all tested biomarkers. For BHB the log likelihood ratio of the final model was χ^2^(17) = 47.55, *p* < 0.001. Two peaks of BHB concentration were observed throughout the fitted period. The first peak occurred directly after parturition, whereas the second occurred between 20 to 40 days p.p. Furthermore, the BHB concentration in the SCK group remained higher compared to the control group until 80 days p.p. For NEFA the log likelihood ratio of the final model was χ^2^(17) = 108.80, *p* < 0.001. In the SCK group, one peak of NEFA concentration was observed directly after parturition followed by a second peak around 40 days p.p. In the control group, one peak was observed after parturition and followed by a continuous decline until the end of the study period. For tBIL the log likelihood ratio of the final model was χ^2^(17) = 85.53, *p* < 0.001. In both groups one peak of tBIL concentration was observed directly after parturition. For AST the log likelihood ratio of the final model was χ^2^(17) = 166.79, *p* < 0.001. In both groups one peak of AST concentration was observed directly after parturition. Subsequently, AST levels declined in both groups. After 30 days p.p., AST concentration of the control group steadily increased in the remaining study period. Compared to the control group, in SCK cows the BHB and tBIL concentrations were significantly higher (*p* < 0.5) from 9 days a.p. until 62 days p.p. and from 2 days p.p. until 62 days p.p., respectively. Detailed results of time intervals showing significantly higher biomarker concentrations are presented in Appendix A.

**Figure 4:**
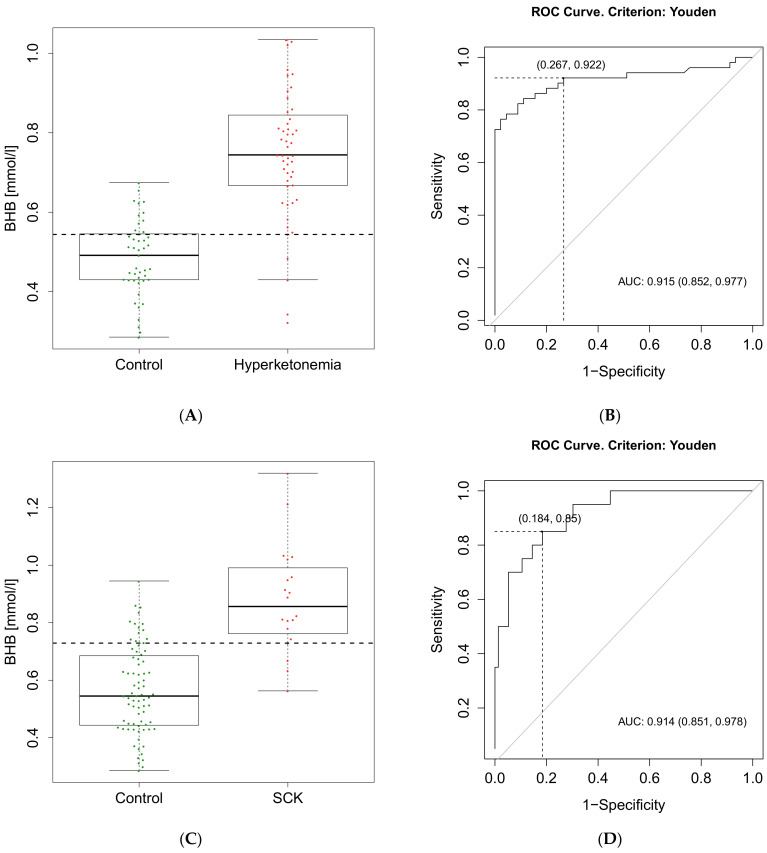
Data on the prognostic classifier for hyperketonemia and subclinical ketosis. (**A**) Boxplot of beta-hydroxybutyrate (BHB) concentrations on the 10th day p.p. for control (BHB ≤ 0.8 mmol/L) and hyperketonemia group (BHB > 0.8 mmol/L); (**C**) Boxplot of BHB concentrations on the 10th day p.p. for control (BHB ≤ 1.2 mmol/L) and SCK group (BHB > 1.2 mmol/L). Dots represent individual measurements. Dashed lines indicate calculated cut-off value (BHB = 0.54 mmol/L) for hyperketonemia and (BHB = 0.73 mmol/L) for SCK classification with the highest area under the curve (AUC); Receiver operating characteristic (ROC) curve for BHB concentration on the 10th day p.p. for hyperketonemia (**B)** and SCK (**D**). Optimal cut-off point was calculated based on the Youden’s index. AUC: area under the curve.

The classifier achieving the highest AUC (AUC = 0.915) for hyperketonemia was the BHB concentration on the 10th day p.p. (t1). A boxplot of BHB concentration at t1 for the hyperketonemic and control groups is shown in Figure 4A. The corresponding ROC curve is presented in Figure 4B. The BHB cut-off value for hyperketonemic classification was calculated to be 0.54 mmol/L. This criterion resulted in 73% specificity (Sp) and 92% sensitivity (Se). The classifier achieving the highest AUC (AUC = 0.914) for SCK was the BHB concentration on the 10th day p.p. (t1). A boxplot of BHB concentration at t1 for the SCK and control groups is shown in Figure 4C. The corresponding ROC curve is presented in Figure 4D. The BHB cut-off value for SCK classification was calculated to be 0.73 mmol/L. This criterion resulted in 82% Sp and 85% Se. A summary of the classification parameters is shown in Table 1. The random decision tree analysis for hyperketonemic and SCK showed consistent results compared to the single classifier analysis; the combination of the different features did not improve the classification.

## 4. Discussion

This study investigated the serum biomarker profile of hyperketonemia and SCK in Simmental dairy cows and prognostic classifiers for hyperketonemia and SCK. A long-term effect of hyperketonemia and SCK was observed on the concentration and time course of BHB. Additionally, a short-term effect of hyperketonemia and SCK after parturition was found in NEFA, tBIL and AST. 

To our knowledge, this study is the first to investigate the long-term effects of hyperketonemia and estimate a cut-off point of BHB concentration in serum to potentially prevent long-term effects of hyperketonemia in dairy cows. 

Several studies were conducted to evaluate a cut-off value for the diagnosis of SCK and its causal association with diseases and infections [9,10,25,27,28,29]. The authors found that a wide range of cut-off levels (>1.00 mmol/L up to >1.6 mmol/L) during the first 2 weeks p.p. were associated with decreased pregnancy rates, uterine infections and displaced abomasum. In our study, hyperketonemic and SCK cows showed higher biomarker concentrations after parturition in BHB, NEFA, tBIL, and AST. This effect might indicate increased metabolic stress during early lactation for ketotic cows [14]. Moreover, Djoković et al. [30] found higher BHB and NEFA concentrations in Simmental dairy cows during the first month of lactation. Increased levels of BHB and NEFA in early lactation might derive from a negative energy balance, leading to overstimulated fat mobilization [4,31,32]. Leblanc et al. [33] described an earlier NEFA increase with greater magnitude in cows experiencing metabolic diseases. However, data in our study showed a greater magnitude of increase, but the increase did not start earlier. Moreover, elevated AST and tBIL concentrations during the first weeks p.p. in hyperketonemic and SCK cows could indicate liver dysfunction [14,22,34,35,36].

Long-term biomarker effects in ketotic Holstein dairy cows were shown previously with elevated BHB levels (BHB > 1.4 mmol/L) after 4 and 8 weeks p.p. [37]. Our results are in agreement with this finding. Furthermore, they revealed that once BHB concentration exceeded 0.8 mmol/L, the further BHB course differed significantly from the course of the control group. Moreover, the BHB concentration did not converge again with the level of the control group until the end of our study period. Hence, an altered BHB course is at least present until the peak lactation phase (80 days p.p.), which supports our main hypothesis. Further studies must be conducted to evaluate whether the changes in BHB concentration persist until the next lactation cycle, and if they are associated with undesirable long-term effects. 

Although this study was conducted on only a limited number of cows of a single breed with moderate milk yield, our results underline the importance of preventing hyperketonemia rather than managing or treating it for a short-term period. However, to our knowledge there are no breed-specific cut-off levels for the biomarkers used in this study. Benedet et al. [38] found small differences (i.e., BHB = 0.65 mmol/L in Holstein versus 0.63 mmol/L in Simmental dairy cows) in concentration, but the biomarker course followed the same biomarker profile over time. We are aware that the chosen cut-off values for this study were very conservative. However, we want to raise the questions: Why were the cut-off values for metabolic biomarkers increased over time? Did our cows change and adapt their metabolism to the increased metabolic stress, or did we push their limits until metabolic diseases arose? Hence, we are claiming that the current gold standard BHB cut-off values of 1.2 mmol/L and 1.4 mmol/L during the first 2 weeks p.p. are too high to prevent the long-term effects of hyperketonemia. As Suthar et al. [27] have already stated, disease-specific thresholds for BHB are widely discussed in research but are not feasible in practice. In our study, hyperketonemia was classified by a BHB concentration > 0.8 mmol/L. As Duffield et al. [10] have already noted, a decision for a cut-off level cannot be an arbitrary choice, but should be a reflection of production and health impairment. Moreover, by raising the cut-off from 0.62 to 0.8 mmol/L, we considered the increased metabolic stress of dairy cows during the transition period [12], as Fürll [8] associates a BHB level > 0.85 mmol/L on the third day p.p. in combination with other parameters as an indicator for increased fat mobilization and FCS. This is also supported by Pralle et al. [39], who found a mean BHB value of 0.8 mmol/L (SD = 0.02) in 1013 Holstein dairy cows. Therefore, it provides feasibility in practice and still reflects individual health status.

The average incidence of SCK is about 40% [40,41]. In the present study the incidence of SCK was lower (20%) but within the expected range. Thirty percent of SCK cases were found within 10 days p.p., wherein most new cases were observed on the fifth day p.p. These results are in accordance with previous findings by Geishauser et al. [41]. Based on the used criteria, we found a hyperketonemia incidence of 52%, wherein 63% of cases were found until the 10th day p.p. These results are in accordance with Duffield et al. [42] and Oetzel [43]. Furthermore, this means that 47% (measurement on sixth day) and 37% (measurement on 10th day) of cases progressing to hyperketonemia are not detected at this stage and would not be detected by a single-day measurement regime, thus pointing out the need for a prognostic biomarker to enable effective preventative action early on. With our model of biomarker progression, such a prognostic classifier was defined. The prognostic classifier for hyperketonemia was 0.54 mmol/L serum BHB measured on the 10th day p.p. This suggests that in contrast to a fixed cut-off level measurable at different time points, a lower cut-off level at the right time point might be superior in the prognosis of hyperketonemia. Such a cut-off level is also supported by the physiological BHB levels for adult cows, published by Fürll [8], who suggested an upper control level of 0.53 mmol/L BHB. For cows exceeding the cut-off, we recommended anti-ketogenic food supplementary treatment such as PG and glycerin. The anti-ketogenic properties of PG and glycerin were shown in various studies, and they are recommended as prophylactic treatment for SCK [44,45,46]. These guidelines might allow increased success in transition management and improved individual health. 

## 5. Conclusions

The longitudinal biomarker profile of a mild form of hyperketonemia was investigated. Our results indicate that the progression of hyperketonemia is closely associated with the biomarker course of BHB, NEFA, tBIL, and AST. The most discriminative biomarker was BHB, which persisted at least until the peak lactation phase (80 days p.p.). The best prognostic classifier for hyperketonemia was identified as a cut-off level of 0.54 mmol/L on the 10th day p.p., achieved with a single measurement regime. This allows effective and early detection of hyperketonemia and is a promising approach for prevention, increasing the overall health of dairy cows.

## Figures and Tables

**Table 1:** Summary of parameters for the calculated cut-off value for hyperketonemia and subclinical ketosis (SCK). The optimal criterion represents the cut-off value resulting in zero false negative cases. BHB: beta-hydroxybutyrate; CI: confidence interval; Se: sensitivity; Sp: specificity; PPV: positive predicted value; NPV: negative predicted value; FP: false positive; FN: false negative; TP: true positive; TN: true negative.

	Hyperketonemia	SCK
Calculated cut-off value of BHB	0.54 mmol/L	0.73 mmol/L
Se (95% CI)	0.92 (0.81–0.98)	0.85 (0.62–0.97)
Sp (95% CI)	0.73 (0.58–0.85)	0.82 (0.71–0.90)
PPV (95% CI)	0.80 (0.66–0.94)	0.55 (0.42–0.87)
NPV (95% CI)	0.89 (0.75–0.95)	0.95 (0.86–0.98)
FP	12	14
FN	4	3
TP	47	17
TN	36	65

**Figure A1:**
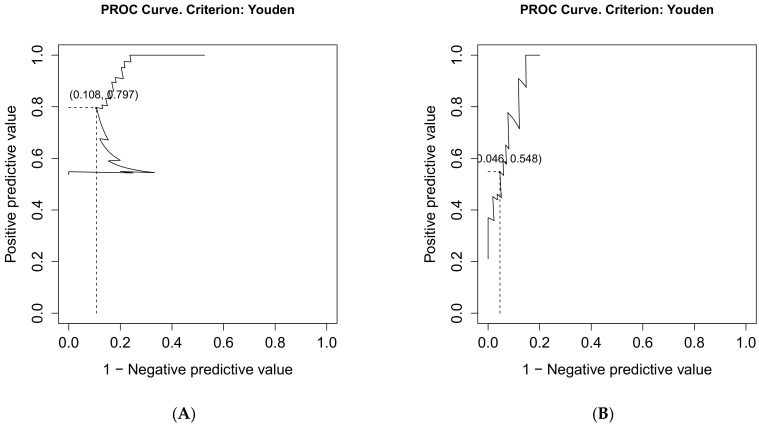
Predictive receiver operation characteristic (PROC) curve for beta-hydroxybutyrate concentration (BHB) on the tenth day post partum for hyperketonemia (**A**) and subclinical ketosis (**B**). Optimal cut point was calculated based on the minimization of the Youden’s index.

## Data Availability

The original contributions generated for the study are included in the article. Further inquiries can be directed to the corresponding author.

## Appendix A

**Table A1:** Summary of *t*-test analysis to evaluate the difference in biomarker concentration in hyperketonemia and subclinical ketotic cows compared to control group. BHB: beta-hydroxybutyrate; NEFA: non-esterified fatty acids; tBIL: total bilirubin; AST: aspartate aminotransferase

	Hyperketonemia				Subclinical Ketosis			
	BHB	NEFA	tBIL	AST	BHB	NEFA	tBIL	AST
15–9 days a.p.	-	-	*p* = 0.02	-	-	-	*p* = 0.04	-
9–3 days a.p.	*p* = 0.02	-	-	-	*p* = 0.04	-	-	-
2–8 days p.p.	*p* < 0.001	-	*p* < 0.01	-	*p* < 0.001	*p* = 0.02	*p* = 0.001	-
8–14 days p.p.	*p* < 0.001	-	*p* < 0.001	-	*p* < 0.001	*p* < 0.01	*p* < 0.001	-
14–20 days p.p.	*p* < 0.001	*p* < 0.01	*p* < 0.001	*p* = 0.04	*p* < 0.001	*-*	*p* = 0.001	*-*
20–26 days p.p.	*p* < 0.001	-	*p* < 0.01	-	*p* < 0.001	*p* < 0.001	*p* < 0.01	-
26–32 days p.p.	*p* < 0.001	-	*p* = 0.01	-	*p* < 0.001	*p* = 0.01	*p* = 0.01	-
32–38 days p.p.	*p* < 0.001	-	*-*	-	*p* < 0.001	*p* < 0.001	*p* < 0.01	-
38–44 days p.p.	*p* < 0.001	-	*p* = 0.02	-	*p* = 0.001	*p* < 0.01	*p* = 0.02	-
44–50 days p.p.	*p* < 0.001	-	-	-	*p* < 0.001	*p* < 0.001	*p* < 0.01	-
50–56 days p.p.	*p* < 0.001	-	-	-	*p* < 0.01	*p* < 0.001	*p* < 0.01	-
56–62 days p.p.	*p* < 0.001	-	-	-	*p* < 0.001	-	*p* = 0.04	-
t0 (day of partum)	*p* < 0.001	-	-	-	*p* < 0.001	*p* = 0.04	*p* < 0.05	-
t10 (10 days p.p.)	*p* < 0.001	*p* < 0.01	*p* < 0.001	-	*p* < 0.001	*p* < 0.01	*p* < 0.001	-
